# Prevalence and determinants of soil transmitted helminth infections among children on Highly Active Antiretroviral Therapy (HAART) in Mulobezi and Sesheke Districts, Zambia: A cross – sectional study

**DOI:** 10.1371/journal.pone.0356060

**Published:** 2026-08-13

**Authors:** Benjamin Mbonshi, Evans Mwape Kabemba, Collins Bowa

**Affiliations:** 1 Department of Biomedical Sciences, School of Health Sciences, The University of Zambia, Lusaka, Zambia; 2 The Department of Clinical Studies, School of Veterinary Medicine, University of Zambia, Lusaka, Zambia; 3 Laboratory Department, Malawi Blood Transfusion Service, Lilongwe, Malawi; University of Uyo, NIGERIA

## Abstract

**Background:**

Soil-transmitted helminth (STH) infections are a major public health challenge in regions where they overlap with HIV/AIDS. Children with HIV may be especially vulnerable to STHs due to immunosuppression, yet the burden and drivers of this co-infection among those on antiretroviral therapy (ART) in Zambia are not well documented. This study aimed to determine the prevalence and identify the key determinants of STH infections among ART-treated children in Western (Mulobezi and Sesheke districts) Zambia.

**Methods:**

We conducted a cross-sectional study with a retrospective clinical review in the Mulobezi and Sesheke districts. From a total population of 487 eligible children, a sample of 103 ART-initiated children aged 5–15 years was enrolled. Participants were selected using stratified random sampling: the sample was first proportionally allocated to six health facilities (strata), and then individual children were randomly selected from clinic lists within each facility. Data was collected using questionnaires, medical records, and laboratory analysis of blood and stool samples (using the Kato-Katz technique). Bivariate analyses used Chi-square/Fisher’s exact and Mann-Whitney U tests. Viral load was log₁₀-transformed for analysis. Variables significant at *p* < 0.1, along with key clinical factors, were entered into a multivariable logistic regression model built via backward stepwise elimination to identify independent predictors. Model fit was assessed using the Hosmer-Lemeshow test.

**Results:**

The prevalence of any STH infection was 11.7% (12/103), with *Ascaris lumbricoides* (6.8%) being the most common species. In the final adjusted model, independent predictors of STH infection were a lower CD4 + count (aOR=3.25 per 100 cells/mm³ decrease; 95% CI: 1.82–5.80), a higher log₁₀ viral load (aOR=2.40 per unit increase; 95% CI: 1.45–3.96), and suboptimal ART adherence, with occasional misses conferring the highest risk (aOR=15.82; 95% CI: 2.98–84.02) compared to very consistent adherence. Lack of clean water access was not retained in the final model.

**Conclusion:**

A notable prevalence of STH infection persists among ART-treated children in this setting, driven primarily by immunological and treatment-adherence factors. These findings support integrating routine STH screening and targeted deworming into paediatric HIV care programs, alongside interventions to optimize ART adherence and immunological recovery.

## Introduction

Soil-transmitted helminths (STHs)—including *Ascaris lumbricoides*, *Trichuris trichiura*, and hookworms (*Necator americanus*, *Ancylostoma duodenale*)—affect over a billion people globally, primarily in low-income regions [1,2]. These infections contribute to a spectrum of clinical effects, including anemia, malnutrition, stunted growth, and impaired cognitive development, which perpetuate cycles of poverty and disease [1,2]. The public health impact is substantial, with infections linked to an estimated 1.38 million disability-adjusted life years (DALYs) worldwide [3].

In Zambia, STH infections are endemic, with prevalence rates exceeding 50% in some regions [4]. School-aged children (5–15 years) are the most affected demographic globally, and this holds true in Zambia, where estimates suggest prevalence rates of 10–40% for *A. lumbricoides*, 1–30% for *T. trichiura*, and hookworms among children [5–7]. Transmission is driven by environmental and socioeconomic factors common in rural areas, such as inadequate sanitation, limited access to clean water, and poverty, which facilitate the contamination of soil with infectious eggs and larvae [7,8].

In sub-Saharan Africa, the high prevalence of STHs frequently overlaps with the burden of HIV/AIDS, with Zambia experiencing persistently high rates of both [9]. This co-occurrence presents unique clinical challenges due to bidirectional immunological interactions. Helminth infections can polarize host immune responses toward a T-helper 2 (Th2) profile, potentially dampening the cell-mediated immunity crucial for controlling HIV and accelerating its progression [10,11]. Conversely, HIV-induced immunosuppression can impair the host’s ability to clear helminths, potentially increasing susceptibility to STH acquisition and worsening their clinical severity [12,13]. For HIV-infected children, the clinical consequences of STHs—such as malnutrition and anemia—can further weaken the immune system, potentially reducing the efficacy of antiretroviral therapy (ART) and increasing the risk of opportunistic infections [14].

Antiretroviral therapy (ART) significantly improves survival and immune function in HIV-infected individuals [15]. However, evidence on ART’s specific impact on STH susceptibility remains inconclusive [13,16]. While immune recovery from ART might be expected to reduce STH risk, determinants of infection among children on ART are multifactorial. Known global and local risk factors persist, including environmental exposures (lack of clean water, poor sanitation), socioeconomic status (poverty, overcrowding), and behavioral factors (hygiene practices) [8,17]. Furthermore, clinical factors unique to this population, such as the degree of immune reconstitution (CD4 + count), virological control (viral load), and ART adherence, may modulate individual risk but are poorly characterized [13,16].

Although studies from other sub-Saharan African countries have examined STH infections among children living with HIV receiving ART, findings are context-specific and cannot be directly generalized to Zambia because of differences in environmental conditions, HIV epidemiology, deworming strategies, and health systems. In Zambia, studies investigating STH infections among ART-treated children, particularly those aged 5–15 years who are frequently excluded from routine deworming programs (like child health week), remain scarce. Consequently, the prevalence and determinants of STH infection in this vulnerable population are poorly characterized, representing an important knowledge gap. Therefore, this study assessed the prevalence of STH infection and its determinants among ART-treated children in Western Zambia to generate locally relevant evidence for integrated HIV-STH control strategies.

## Methods

### Study design and setting

A hybrid study design was employed. A cross-sectional component was used to determine the point prevalence of STH infections and collect concurrent data on potential risk factors among the study participants. A retrospective review component was utilized to extract clinical data (e.g., CD4 + count trends, viral load history, deworming records) from patient files. This hybrid approach provided a comprehensive snapshot of current infection status while contextualizing it within the participants’ clinical history, which is pertinent for understanding STH determinants in a chronic care population.

The study was conducted from July 10 to July 31, 2024, across six health facilities in the Mulobezi and Sesheke districts of Western Zambia. These districts were purposively selected due to their: (1) classification as highly endemic for STH infections [4]; (2) high HIV/AIDS prevalence [18]; and (3) shared coverage under integrated donor-supported HIV/TB programs, which facilitated access to standardized health records. The setting features a seasonal tropical climate conducive to environmental transmission of STHs, with a warm, rainy season (November-April) and a dry season (May-October), average temperatures of 25–30°C, and vegetation dominated by Miombo and Kalahari woodlands [19]. Mulobezi (population 45,326) is rural with dispersed settlements, while Sesheke (population 72,655) is peri-urban, allowing for the examination of varying environmental and socioeconomic determinants [20].

### Participants and sampling technique

Eligible participants were children aged 5–15 years who had been receiving Antiretroviral Therapy (ART) for at least six months and were residents of the study districts. A resident was defined as a child who had continuously lived within the catchment area of the participating health facility for at least six months preceding enrolment. Residency status was confirmed through guardian report and cross-checked against health facility records. Children presenting with severe illness requiring immediate medical attention, hospital admission, or whose clinical condition prevented participation in the interview or provision of biological specimens were excluded. Children who had received deworming medication within the three months preceding data collection were also excluded because recent anthelmintic treatment could suppress detectable helminth egg excretion, resulting in false-negative stool examinations that may not accurately reflect the underlying infection status. The three-month exclusion period was therefore adopted to minimize misclassification of current infection status. Additional exclusion criteria included refusal of parental or guardian consent, refusal of child assent where applicable, inability to provide a stool specimen after reasonable follow-up, incomplete questionnaire or laboratory data required for the primary analysis, and non-residency within the study area.

The sample size was calculated using the Cochran formula for a finite population. With an estimated STH prevalence of 14.4% from a previous Zambian study [7], a 95% confidence level, a 5% margin of error, and a total eligible population (N) of 487 children, the calculated sample size was 136. A stratified random sampling technique was employed, with the six participating health facilities serving as the sampling strata. The sample size was proportionally allocated to each facility according to the number of eligible children receiving ART. Within each facility, a sampling frame was developed from clinic appointment registers containing all eligible children. Each eligible child was assigned a unique identification number, after which simple random sampling was performed using computer-generated random numbers to select participants until the required sample size for each facility was attained. This approach ensured that every eligible child had an equal probability of selection while maintaining proportional representation across the participating health facilities.

### Data collection and management

#### Questionnaire administration.

A structured interviewer-administered questionnaire was used to collect socio-demographic, environmental (e.g., water source and sanitation), and behavioral information. Interviews were primarily conducted with parents or legal guardians because they were considered the most reliable source of information regarding household socioeconomic characteristics, environmental exposures, and the child’s medical history. However, older children who provided assent were invited to respond to age-appropriate questions, particularly those relating to their personal hygiene practices and behaviors, while their responses were corroborated with information provided by their guardians where necessary. Interviews were conducted in private rooms within the health facilities, separate from the general waiting area, to ensure privacy and minimize stigma. Data were collected by trained research assistants with health science backgrounds under the supervision of the principal investigator. Clinical information, including ART regimen, treatment adherence (based on pharmacy refill records and self-report), and deworming history, was extracted from patient medical records and supplemented with questionnaire data where appropriate.

#### Biological sample collection and analysis.

*Blood Samples*: Approximately 4mL of venous blood was collected per participant. Full blood count (FBC) analysis was performed on EDTA samples using an ABX Micros analyzer. CD4 + T-cell counts were determined using either the BD FACSCount system or the Pima CD4 + analyzer via flow cytometry. HIV viral load quantification was performed using PCR technology on the Roche Cobas® Ampliprep or Gene Xpert systems, utilizing records from tests within the prior six months where available to avoid redundancy.

*Stool Samples*: Participants and their guardians received standardized verbal instructions on the correct procedure for stool specimen collection before sample collection. They were instructed to collect approximately 5–10 g of freshly passed stool directly into a pre-labelled, leak-proof container while avoiding contamination with urine, water, or soil. Samples were transported in cool boxes and processed within hours of collection. The Kato-Katz technique was employed for microscopic examination. A standardized 41.7 mg stool sample was prepared on a glycerol-malachite green slide and examined after a clearing period of 30–60 minutes. Helminth eggs were identified according to standard morphological characteristics [21]. All slides were independently examined by two experienced microscopists. Where discrepant findings occurred, defined as disagreement in positivity or an egg count difference exceeding 10%, the slide was reviewed by a senior biomedical laboratory technologist, whose reading was considered final.

#### Data management.

All questionnaire, laboratory, and clinical data were entered into a customized database using PSPP software. Data cleaning involved range checks, consistency checks, and verification against a randomly selected 10% sample of the original data collection forms. Laboratory results were entered directly from source reports to minimize transcription errors.

### Statistical analysis

Data were analyzed using PSPP version 1.6.2. Descriptive statistics were computed for all study variables. Categorical variables were summarized as frequencies and percentages, while continuous variables were assessed for normality. As key clinical variables were not normally distributed, they were summarized using medians and interquartile ranges (IQRs; 25th–75th percentiles).

Bivariate analysis was performed to identify factors associated with the primary outcome of STH infection (present/absent). Pearson’s Chi-square test was used when all expected cell frequencies were at least five, whereas Fisher’s exact test was applied when one or more expected cell frequencies were less than five. Continuous variables were compared using the Mann-Whitney U test. Owing to its highly skewed distribution, plasma viral load (copies/mL) was log₁₀-transformed before analysis; values reported as “target not detected” were imputed as one-half of the assay’s lower limit of detection before transformation.

Variables with a *p*-value < 0.10 in the bivariate analysis, together with clinically relevant variables (CD4 + count and viral load), were entered into a multivariable binary logistic regression model to identify independent determinants of STH infection. The final model was developed using backward stepwise elimination with a retention criterion of *p* < 0.05. Adjusted odds ratios (aORs) and their corresponding 95% confidence intervals (CIs) were reported. Model fit was assessed using the Hosmer-Lemeshow goodness-of-fit test. A two-sided *p*-value < 0.05 was considered statistically significant.

### Quality control

The questionnaire was pre-tested among approximately 10% of the calculated sample size at a health facility with characteristics similar to those of the study sites but not included in the main study. The pre-test was conducted by the research team to assess the clarity, sequence, comprehensibility, and completeness of the questionnaire, and participants involved in the pre-test were not included in the final study sample. Before commencement of data collection, the principal investigator conducted a two-day training workshop for research assistants covering the study objectives, participant recruitment procedures, informed consent and assent, questionnaire administration, confidentiality, data recording, and specimen handling. Laboratory analyses were performed by experienced biomedical laboratory technologists routinely involved in parasitological diagnosis. Prior to the study, they received orientation on the study protocol, standardized Kato-Katz procedures, specimen handling, quality assurance, and recording procedures to ensure consistency across study sites. Standard operating procedures were followed throughout the study, and positive and negative controls were included with each laboratory batch where applicable.

### Ethical approval

Ethical approval was obtained from the University of Zambia Biomedical Research Ethics Committee (Ref: UNZABREC FWA00000338; IRB00001131) and the National Health Research Authority. Permission to conduct the study was granted by the District Health Directors of Mulobezi and Sesheke districts. Before enrolment, written informed consent was obtained from the parents or legal guardians of all participating children. In addition, written assent was obtained from children who were capable of providing assent in accordance with national ethical guidelines. Confidentiality was maintained throughout the study by using unique study identification numbers instead of participant names.

## Results

### Demographic characteristic of participants

A total of 103 children living with HIV (CLWH) aged 5–15 years participated. The mean age was 11.8 years (SD = 2.3), with balanced representation from Mulobezi (43.7%) and Sesheke (56.3%). Most participants were male (61.2%). Age distribution was skewed toward older children (9–15 years: 87.4%) (Table 1).

**Table 1:** Demographic characteristics of children on ART in Muobezi and Sesheke districts, Zambia (N = 103).

Characteristic	Category	Frequency (n)	Percentage (%)
**District**	Mulobezi	45	43.7
	Sesheke	58	56.3
**Age group**	5–8 years	13	12.6
	9–12 years	39	37.9
	13–15 years	51	49.5
**Gender**	Male	63	61.2
	Female	40	38.8

### Prevalence of STH infections and parasitological findings

The overall prevalence of soil-transmitted helminth (STH) infection was 11.7% (12/103; 95% CI: 6.8–19.8%). *Ascaris lumbricoides* was the predominant species, with a prevalence of 6.8%, followed by hookworm (2.9%), *Trichostrongylus* spp. (1.0%), and *Strongyloides stercoralis* (1.0%) (Table 2). The prevalence of **any STH infection** did not differ significantly between the two study districts (Mulobezi: 13.3% vs. Sesheke: 10.3%; Fisher’s exact test, *p* = 0.760).

**Table 2:** Species-specific prevalence of soil-transmitted helminths among participants (N = 103).

Species	Cases (n)	Prevalence (%)	95% CI
*Ascaris lumbricoides*	7	6.8	3.3–13.4
Hookworm	3	2.9	1.0–8.2
*Trichostrongylus* spp.	1	1.0	0.2–5.3
*Strongyloides stercoralis*	1	1.0	0.2–5.3
**Any STH infection**	**12**	**11.7**	**6.8–19.8**

### Bivariate Analysis of Factors associated with STH infections among children living with HIV on ART

No statistically significant associations were observed between STH infection and guardian’s education level (*p* > 0.05), primary household income source (*p* > 0.05), type of sanitation facility (*p* > 0.05), handwashing practices (*p* > 0.05), or dietary habits (*p* > 0.05). However, children from households without access to a clean water source were significantly more likely to have an STH infection than those with access to clean water (75.0% vs. 3.3%; *p* = 0.011) (Table 3).

**Table 3:** Bivariate Analysis of Factors associated with STH infections among children living with HIV on ART.

Factor	STH Positive (n = 12)	STH Negative (n = 91)	*p*-value
**Immunological (Median [IQR])**			
CD4 + count (cells/mm³)	402 [250.5–619.75]	782 [650–860]	0.001
Viral load (copies/mL)	1437.5 [905.5–2657]	22 [0–166]	<0.001
**Environmental (n (%))**			
Lack of clean water access	9 (75.0)	3 (3.3)	0.011
**Clinical & Behavioural (n (%))**			
**ART Adherence**			<0.001
Occasional misses	7 (58.3)	1 (1.1)	
Mostly consistent	4 (33.3)	16 (17.6)	
Very consistent	1 (8.3)	74 (81.3)	
Presence of helminth-related symptoms	7 (58.3)	5 (5.5)	0.015

**Note:** IQR, Interquartile Range. The *p*-value for ART adherence is for the overall comparison across categories. For Viral load Mann-Whitney U test performed on log₁₀-transformed values.

Clinical and immunological characteristics also differed significantly according to STH infection status. Children with STH infection had a significantly lower median CD4 + count than those without infection (402 [IQR: 250.5–619.75] vs. 782 [IQR: 650–860] cells/mm³; *p* = 0.001) and a significantly higher median viral load (1,437.5 [IQR: 905.5–2,657] vs. 22 [IQR: 0–166] copies/mL; *p* < 0.001). ART adherence was significantly associated with STH infection (*p* < 0.001), with infection occurring more frequently among children reporting occasional missed doses than among those with very consistent adherence. The presence of helminth-related symptoms was also significantly associated with a positive stool examination (58.3% vs. 5.5%; *p* = 0.015) (Table 3).

### Multivariable analysis of determinants of STH infection

Following adjustment for potential confounding factors, lower CD4 + count, higher viral load, and suboptimal ART adherence remained independently associated with STH infection (Table 4). A decrease of 100 cells/mm³ in CD4 + count was associated with more than a threefold increase in the odds of STH infection (adjusted OR = 3.25, 95% CI: 1.82–5.80; *p* < 0.001). Similarly, each one-unit increase in log₁₀ viral load was associated with increased odds of STH infection (adjusted OR = 2.40, 95% CI: 1.45–3.96; *p* = 0.001). Compared with children reporting very consistent ART adherence, those reporting occasional missed doses had markedly higher odds of STH infection (adjusted OR = 15.82, 95% CI: 2.98–84.02; *p* = 0.001), whereas mostly consistent adherence was not statistically significant (adjusted OR = 4.12, 95% CI: 0.79–21.43; *p* = 0.093). Although lack of access to a clean water source was associated with STH infection in the bivariate analysis, it did not remain an independent predictor after multivariable adjustment. The final model demonstrated good calibration (Hosmer-Lemeshow goodness-of-fit test, *p* = 0.492) (Table 4).

**Table 4:** Adjusted multivariable logistic regression analysis of factors associated with STH infections.

Variable	Adjusted OR	95% CI	*p*-value
**HAART adherence** (Ref: Very consistent)			
Mostly consistent	4.12	0.79–21.43	0.093
Occasional misses	15.82	2.98–84.02	0.001
**CD4 + count** (per 100 cells/mm³ increase)	3.25	1.82–5.80	<0.001
**Viral load** (per log10 increase)	2.40	1.45–3.96	0.001

Abbreviations: OR, odds ratio; CI, confidence interval; Ref, reference category.

### Post study actions

All participants diagnosed with an STH infection during the study were managed according to national guidelines. Their primary healthcare providers at the respective study health facilities were notified immediately for treatment and follow-up, ensuring continuity of care within the existing HIV/ART clinical service structure.

## Discussion

This study documents an 11.7% prevalence of soil-transmitted helminth (STH) infections among children receiving antiretroviral therapy (ART) in Western Zambia. This finding indicates a persistent burden of parasitic infection in a paediatric HIV care population, despite ongoing ART services. When compared with evidence from HIV-infected populations elsewhere, our prevalence is broadly consistent with pooled estimates reported in people living with HIV, such as the 8% prevalence of *Ascaris lumbricoides* reported by Akanksha et al. (2023) [22,23], which is comparable to the 6.8% observed in this study. Similarly, studies conducted in sub-Saharan Africa, including settings within Zambia and neighboring countries, have reported variable STH prevalence among HIV-infected children, generally ranging from low to moderate levels. These variations are likely attributable to differences in environmental exposure, deworming coverage, sanitation conditions, and intensity of HIV care implementation across settings.

The predominance of *Ascaris lumbricoides* (6.8%) observed in this study is consistent with its known epidemiological dominance in tropical and subtropical regions where environmental conditions favor transmission. In this context, the distribution of STH species is primarily driven by ecological and behavioral factors such as soil contamination, sanitation practices, and water hygiene. However, it is also important to consider that HIV-related immunosuppression may influence the intensity and persistence of helminth infections rather than the species distribution itself. Reduced CD4 + T-cell function may impair effective immune-mediated parasite clearance, potentially allowing higher worm burdens once infection is established, although it is not considered a primary determinant of species predominance. Therefore, the observed pattern likely reflects a combination of strong environmental transmission pressure in endemic areas alongside host immune status, rather than HIV infection selectively favoring *Ascaris* acquisition.

Comparatively, the species distribution in this study aligns with findings from similar paediatric studies in endemic African settings, where *Ascaris lumbricoides* is consistently the most frequently identified STH, followed by hookworm and, less commonly, *Trichuris trichiura* and *Strongyloides stercoralis*. For example, studies in other Zambian rural districts and comparable settings have reported *Ascaris* as the dominant species among school-aged children, largely attributed to its high environmental resilience and low infectious dose requirement. The relatively low prevalence of hookworm and *Strongyloides stercoralis* in this study is also consistent with reports from comparable ART cohorts, although even low levels of *Strongyloides* remain clinically important due to its potential severity in immunocompromised hosts. Differences in species distribution between studies may be explained by variations in diagnostic sensitivity, local sanitation infrastructure, climate conditions, and the frequency of mass deworming interventions.

The finding that *Strongyloides stercoralis* and hookworm infections were present, although at low prevalence, is clinically relevant in this immunocompromised population. *Strongyloides stercoralis*, in particular, warrants attention due to its potential for autoinfection and severe disease in individuals with impaired cellular immunity. Similarly, hookworm infection remains important due to its contribution to chronic blood loss and anaemia, which may compound existing HIV-related haematological complications. In this regard, the species-specific profile observed in this study highlights that even a relatively modest overall prevalence can still represent meaningful clinical risk when considered at the parasite level and within an immunocompromised host population. This interpretation is directly supported by the observed distribution of species and their known clinical implications within HIV-infected children in this study setting.

The associations observed between STH infection and lower CD4 + counts, higher viral load, and suboptimal ART adherence reflect relationships between immune status, treatment effectiveness, and infection risk. These findings suggest that children with poorer immunological recovery and virological suppression had higher odds of STH infection. However, these associations should be interpreted as correlational rather than causal, given the cross-sectional nature of the study. It is also important to note that ART effectiveness, reflected by viral suppression and immunological recovery, may serve as an indirect marker of overall health status and healthcare engagement, which could also influence exposure and susceptibility to infections.

Although environmental and socioeconomic factors such as access to clean water were significant at bivariate level, they did not remain independently associated in the multivariable model. This may suggest that within this ART-engaged cohort, individual-level clinical factors related to HIV disease control may have a stronger measurable association with STH infection than household-level variables captured in this study. However, this does not diminish the established importance of Water, Sanitation and Hygiene (WASH) interventions in STH prevention, as highlighted in previous evidence [24]. Rather, it may reflect the relatively homogeneous exposure environment in endemic rural and peri-urban settings, where baseline environmental risk is high across most households.

In terms of implications, the persistent prevalence observed in this study suggests that routine deworming alone may not fully address STH burden in children living with HIV. Integrating targeted screening strategies into routine HIV care may be beneficial, particularly for children with poor immune recovery or unsuppressed viral load. Additionally, reinforcing adherence to ART may have broader health benefits beyond HIV control, including reducing vulnerability to opportunistic infections such as STHs. However, these recommendations should be interpreted cautiously and implemented alongside established public health measures such as WASH improvements and periodic deworming programs.

### Limitations

This study’s findings should be interpreted in light of several limitations. The cross-sectional design limits our ability to establish causality between identified risk factors and STH infection. The final sample size, while adequate for estimating prevalence, provided a limited number of STH-positive cases (n = 12), which constrained the number of variables that could be reliably included in the multivariable model and affects the precision of our effect estimates. The use of a single Kato-Katz thick smear, though standard, may have led to an underestimation of true prevalence, particularly for hookworm and *Strongyloides stercoralis*. Furthermore, this study did not reliably measure or classify infection intensity, which is an important parameter for assessing individual morbidity risk and population transmission dynamics. Reliance on self-reported data for adherence and symptoms introduces potential recall and social desirability bias. Other unmeasured confounders, such as detailed nutritional status, specific WASH behaviors, or environmental exposure intensity, could influence risk. Finally, the study’s focus on two high-burden districts may affect the generalizability of findings to other settings in Zambia.

## Conclusion

In conclusion, this study confirms that STH infections remain a notable public health issue among ART-treated children in Western Zambia. The findings identify a distinct risk profile for this clinical cohort, where individual immunological and treatment-adherence factors are paramount. Specifically, compromised immune status (evidenced by a lower CD4 + count and a higher viral load) and suboptimal ART adherence were strong, independent determinants of STH infection.

These findings lead to two primary, actionable recommendations. First, for clinical practice, we recommend the integration of routine STH management into standard paediatric HIV care packages. A practical, risk-stratified approach within ART clinics should prioritize children with poor immunological status or adherence challenges for regular screening and presumptive treatment. Second, for public health policy, the results underscore that effective STH control in this vulnerable population is inextricably linked to optimizing HIV care outcomes. Strengthening ART adherence support to achieve sustained virological suppression and immune recovery represents a critical strategy for reducing parasitic co-infection burden. Future research should employ longitudinal designs to clarify causal pathways and evaluate the impact and cost-effectiveness of integrated, clinic-based screening and management models.

## Supporting information

S1 Data De-identified dataset used for statistical analysis. (XLSX)
